# Second Neoplasms in Italian Patients with Hairy Cell Leukemia after Treatment with Cladribine: A Multicenter Investigation and Literature Review

**DOI:** 10.3390/cancers16081475

**Published:** 2024-04-11

**Authors:** Marianna Criscuolo, Maria Elena Tosti, Alessandro Broccoli, Marzia Varettoni, Alessio Maria Edoardo Maraglino, Antonella Anastasia, Maria Cantonetti, Livio Trentin, Sofia Kovalchuk, Lorella Orsucci, Marina Deodato, Angelica Spolzino, Stefano Volpetti, Ombretta Annibali, Sergio Storti, Caterina Stelitano, Francesco Marchesi, Sonia Morè, Luana Fianchi, Brunangelo Falini, Alessandro Pulsoni, Enrico Tiacci, Pier Luigi Zinzani, Livio Pagano

**Affiliations:** 1 Dipartimento di Diagnostica per Immagini, Radioterapia Oncologica ed Ematologia, Fondazione Policlinico Universitario A. Gemelli IRCCS, 00168 Rome, Italy; marianna.criscuolo@policlinicogemelli.it (M.C.); luana.fianchi@policlinicogemelli.it (L.F.); 2 Istituto Superiore Sanità, 00161 Rome, Italy; mariaelena.tosti@iss.it; 3 IRCCS Azienda Ospedaliero-Universitaria di Bologna, Istituto di Ematologia “Seràgnoli”, 40138 Bologna, Italy; alessandro.broccoli6@unibo.it (A.B.); pierluigi.zinzani@unibo.it (P.L.Z.); 4 Dipartimento di Medicina Specialistica, Diagnostica e Sperimentale Università di Bologna, 40126 Bologna, Italy; 5 Divisione di Ematologia, Fondazione IRCCS Policlinico San Matteo, 27100 Pavia, Italy; m.varettoni@smatteo.pv.it; 6 Oncohematology Division, IEO European Institute of Oncology IRCCS, 20141 Milan, Italy; alessiomariaedoardo.maraglino@ieo.it; 7 Department of Hematology, ASST Spedali Civili, 25123 Brescia, Italy; antonella.anastasia@gmail.com; 8 Dipartimento di Oncoematologia Policlinico Tor Vergata, Università Tor Vergata, 00133 Rome, Italy; cantonet@uniroma2.it; 9 Division of Haematology and Clinical Immunology, University of Padova, 35137 Padua, Italy; livio.trentin@unipd.it; 10 SOD C Ematologia, AOU Careggi, 50134 Firenze, Italy; to.sofia@mail.ru; 11 S.C. Ematologia, A.O.U. Città della Salute e della Scienza di Torino, 10126 Turin, Italy; lorsucci@cittadellasalute.to.it; 12 Divisione di Ematologia, ASST Grande Ospedale Metropolitano Niguarda, 20162 Milan, Italy; marina.deodato@ospedaleniguarda.it; 13 Dipartimento di Medicina e Chirurgia, Università degli Studi di Parma, 43125 Parma, Italy; angelica.spolzino@hotmail.it; 14 Oncoematologia, Istituto Oncologico Veneto IOV-IRCSS, 31033 Castelfranco Veneto, Italy; 15 Clinica Ematologia, Azienda Sanitaria Universitaria Integrata, 33100 Udine, Italy; stefano.volpetti@asuiud.sanita.fvg.it; 16 Hematology and Stem Cell Transplantation Unit, Campus Bio-Medico University, 00128 Rome, Italy; o.annibali@unicampus.it; 17 UOC Oncoematologia Fondazione di Ricerca e Cura Giovanni Paolo II, Campobasso—Università Cattolica del Sacro Cuore, 86100 Campobasso, Italy; profsergiostorti@gmail.com; 18 Divisione di Ematologia, Azienda Ospedaliera “Bianchi Melacrino Morelli”, 89125 Reggio Calabria, Italy; caterinastelitano27@gmail.com; 19 Haematology and Stem Cell Transplantation Unit, IRCCS Regina Elena National Cancer Institute, 00144 Rome, Italy; francesco.marchesi@ifo.it; 20 Clinica di Ematologia, AOU delle Marche, 60126 Ancona, Italy; sonia.more@ospedaliriuniti.marche.it; 21 Department of Medicine and Surgery, Institute of Hematology and Center for Hemato-Oncology Research, University of Perugia, Santa Maria della Misericordia Hospital, 06123 Perugia, Italy; brunangelo.falini@unipg.it (B.F.); enrico.tiacci@unipg.it (E.T.); 22 Divisione di Ematologia, Department of Translational and Precision Medicine, Sapienza University of Rome, 00185 Rome, Italy; alessandro.pulsoni@uniroma1.it; 23 Sezione di Ematologia, Dipartimento di Scienze Radiologiche ed Ematologiche, Università Cattolica del Sacro Cuore, 00168 Rome, Italy

**Keywords:** hairy cell leukemia, cladribine, second cancer

## Abstract

**Simple Summary:**

The prevalence of second neoplasms among patients with hairy cell leukemia (HCL) treated with cladribine is not negligible. Immunosuppression, high rate of cure, and aging may have a role in the pathogenesis of second cancers after HCL. We aimed to compare the risk of cancer between patients with HCL treated with cladribine and the general population.

**Abstract:**

Concern has emerged about the prevalence of second cancers among patients with hairy cell leukemia (HCL) treated with purine analogs. We investigated 513 patients with HCL treated with cladribine over the last 30 years at 18 Italian centers and calculated their standardized incidence ratios (SIRs). We identified 24 patients with a second cancer diagnosed at a median time from treatment with cladribine of 59.9 months (range: 9.2–169.7 months). All patients with solid neoplasms presented with a limited-stage disease, except four cases of locally advanced cancer; multiple myeloma patients had a smoldering disease, while lymphoma patients had stage Ie and stage IV diseases. Response to therapy was complete in 19 cases; 1 patient is still receiving treatment for a relapsing bladder disease, while 2 patients progressed during treatment and died. These two patients died from unrelated causes: one from infection and one due to surgery complications. The median OS from HCL was 98.5 months (range: 38.4–409.2 months), while the median OS from second cancer was 27.6 months (range: 1–117.8 months). The SIR was 0.86 (95% CI: 0.54–1.30) for males and 1.13 (95% CI: 0.36–2.73) for females: no statistically significant differences were highlighted. We were not able to demonstrate an excess of second cancer or a significant association with the specific studied neoplasm.

## 1. Introduction

Hairy cell leukemia (HCL) is a rare lymphoproliferative neoplasm that accounts for less than 2% of all leukemias. Since its first identification in 1923, specific disease characteristics have been established and are currently used for diagnosis and prognostic evaluation. The typical immunophenotype characterized by cell surface positivity for CD19, CD20, CD22, CD11c, CD25, CD103, and CD123 and the presence of the BRAF-V600E mutation are hallmarks of classic HCL [1,2]. The introduction of purine analogs has completely changed the prognosis of HCL patients: a considerably high rate of complete remission (CR) has been reported after a single course of purine analogs (i.e., cladribine and pentostatin). Ever since the first paper on this topic was published in 1990, reporting durable remission after cladribine treatment in previously treated patients with HCL [3], several papers have investigated response rates and long-term remission with cladribine treatment, and the use of different treatment schedules. The CR rates ranged from 60% to more than 90% over the last two decades [4], with a median duration of response of approximately 10 years [5]. Moreover, no differences were reported between continuous infusion, 2 h infusion, and subcutaneous injection in terms of the depth of response or adverse events [5,6,7].

Considering the excellent prognosis of HCL, safety concerns are highly addressed in this population. In recent years, outcomes have improved mostly because of improvements in supportive therapy and infection control, although the infection rate is almost stable [5,6,7,8]. On the other hand, most papers have reported a variable prevalence of second neoplasms among patients treated with cladribine. According to some studies, patients with HCL might be at greater risk of developing a second cancer than the general population, particularly after treatment with purine analogs. It is not clear whether there is an excess of second cancers in this population and whether this may depend on the disease itself, the type of drug used, or its long-term immunosuppressive effect.

In this report, we investigated the prevalence of second neoplasms among HCL patients treated with cladribine over the last 30 years and compared the findings with the prevalence of cancer in the Italian general population.

## 2. Materials and Methods

We gathered demographic, clinical, and laboratory data from 513 evaluable patients from 18 Italian centers. The patients were diagnosed with HCL from March 1991 to May 2019, and all patients were treated with first-line cladribine. The response rate and long-term remission data for this population were published elsewhere [5]. In this report, we focused on the prevalence of second neoplasms after first-line treatment with cladribine. Information about the type and stage of neoplasm, the treatment administration and response, and the outcome was recorded. Subsequently, we calculated the standardized incidence ratios (SIRs) and 95% confidence intervals (95% CIs) for second neoplasms by comparing the observed number of cases to those expected based on the incidence rates recorded among the Italian population in 2020 and based on Italian cancer registries [9]; the Eurostat 2013 population was used for standardization.

This study was approved by the local Ethics Committee, and all patients provided their consent to participate. The study was conducted in accordance with the Helsinki Declaration of 1975, as revised in 2008.

The datasets used and/or analyzed during the current study are available from the corresponding author upon reasonable request.

## 3. Results

We identified 24/513 patients with a second neoplasm diagnosed during a follow-up assessment for their HCL (Table 1). None of these patients had a previous diagnosis of a solid or hematological neoplasm. The median age at HCL diagnosis was 63 years (range: 43–77 years) for these 24 patients, with a strong preponderance of male patients (20/24). The response to HCL treatment was CR in 18 patients, partial (PR) in 4 patients, and hematological improvement (HI) in 2 patients, respectively; these response criteria were used as per recent consensus guidelines [10]. Two patients were re-treated for relapse 26.7 and 51 months before diagnosis of the second neoplasm, respectively, and 65.5 and 11.3 months after achieving CR.

The median time from the end of treatment with cladribine to the time of diagnosis of a second neoplasm was 59.9 months (range: 9.2–169.7 months). During the follow-up for HCL, six patients were diagnosed within 3 years after being treated with cladribine, seven patients and six patients were diagnosed within 3 to 5 years and 5 to 8 years after cladribine treatment, and five patients were diagnosed more than 8 years after cladribine treatment. The median age at diagnosis of a second neoplasm was 72 years (range: 44–84 years). The histologies, stages, and treatments of the second neoplasms are reported in Table 1. All 15 patients responded to therapy after surgery alone, 3 patients responded after combination therapy, and 1 patient responded after chemotherapy. One patient is still receiving treatment for a relapsing bladder disease, while two patients progressed during treatment and eventually died from a second neoplasm.

The median OS from HCL diagnosis was 98.5 months (range: 38.4–409.2 months), while the median OS from the diagnosis of a second neoplasm was 27.6 months (range: 1–117.8 months). Overall, six patients died (25%)—one from infection after multiple relapses of HCL, two from the progression of their second neoplasm (lung cancer and lymphoma), one from an abdominal surgery complication, one from a cardiovascular event, and one from a natural event. Considering the whole population of 513 patients, the median OS was not reached and more than 80% of patients were estimated to be alive after 15 years.

We calculated the standardized incidence of second cancer among the HCL population according to sex: 543.3 × 100,000 (95% CI: 283.6–803.1) for males and 492.6 × 100,000 (95% CI: 0.0–1099.0) for females. The SIRs were 0.86 (95% CI: 0.54–1.30) for the males and 1.13 (95% CI: 0.36–2.73) for the females. Moreover, we compared our results with the standardized rates of neoplasms among the Italian general population according to the 2020 AIRTUM database: 704.4 × 100,000 for males and 484.7 × 100,000 for females. No statistically significant differences were highlighted.

## 4. Discussion

Early after the introduction of the extensive use of cladribine, some reports concerning the prevalence of second neoplasms among patients with HCL were published (Table 2). In a registry-based study published in 1997, Kurzrock reported 26 diagnoses of second neoplasms among 350 patients with HCL treated with IFN, pentostatin, and cladribine [11]. Although a significant excess of plasma cell neoplasms and lymphoma was reported (O/E, 13.04; *p* < 0.001 and O/E, 8.7; *p* = 0.03, respectively), an excess of second neoplasms specifically related to a single drug was not detected (IFN, *p* = 0.27; cladribine, *p* = 0.37; pentostatin, *p* = 0.7).

Since then, the debate about the risk of second cancer after treatment with cladribine has been periodically updated. Au and colleagues reported 117 patients diagnosed with HCL between 1976 and 1996 who were treated according to four clinical trials with various combinations of splenectomy, interferon alpha (IFN), and cladribine, and followed up prospectively [13]. In this population, 28 second cancers were diagnosed in 25 patients (21.3%) at a median of 40 months after their HCL diagnosis. In 2002, a multicenter Italian study specifically designed to assess the incidence of second cancers reported 54 cases of second neoplasms among 1022 patients with HCL [14]. The cumulative risk of second cancer was 5%, 10%, and 14% at 5, 10, and 15 years, respectively. Similarly, a more recent paper reported the results of a retrospective survey on 279 French patients, with a cumulative incidence of second cancer of 15% (95% CI: 11; 19) at 10 years (solid cancer: 11% [95% CI: 7.2; 15] and hematological malignancy (HM): 5% [95% CI: 2.8; 8.2]) [20].

When comparing the risk of second cancer in HCL patients with the incidence of cancer in the general population, the results varied according to different studies. In the paper by Au et al. [13], the overall relative risk was 2.6: in particular, a marked increase in the relative risk was reported for prostate and colon cancers. In another report [14], the overall incidence was not significantly greater than expected (SIR 1.01, *p* = 1.0, 95% CI 0.74 to 1.33), but the standardized incidence ratio (SIR) was up to 5.3% for non-Hodgkin’s lymphoma (95% CI: 1.9 to 11.5). No significant differences were recorded according to previous therapies (no IFN, first-line IFN, or second-line IFN). A large population-based study reported 358 diagnoses of second cancer among 3104 patients with HCL observed from 1973 to 2002 with a median follow-up of 6.5 years, for which the SIR was 1.24 (95% CI: 1.11–1.37) [16]. Importantly, the risk of second cancer was comparable among both the 1509 patients treated with chemotherapy (SIR 1.35, 95% CI 1.13–1.60) and the 1595 patients who did not receive chemotherapy (SIR 1.18, 95% CI 1.03–1.35). The authors reported that the risk was more pronounced for Hodgkin’s and non-Hodgkin’s lymphomas (SIR 6.61, 95% CI 2.13–15.42 and SIR 5.03, 95% CI 3.77–6.58, respectively) and for thyroid neoplasms (SIR 3.56, 95% CI 1.30–7.74). In another population-based study [15], 12 second cancers were diagnosed among 181 patients with HCL mostly treated with cladribine between 1991 and 2001 (6.6%). The SIR was 1.3 (95% CI: 0.68–2.28) overall, with a marked increase in genito-urinary neoplasms (SIR 3.23, 95% CI 1.39–6.36, *p* = 0.008).

In a registry-based study on second primary cancers after acute and chronic lymphoproliferative neoplasms, Zheng et al. reported 150 second cancers among 823 patients with HCL (18.2%) diagnosed from 1986 to 2015 after a median follow-up of 8 years [19]. Overall, the relative risk of second cancer was 1.65; the most significant associations were reported with squamous cell skin carcinoma, kidney carcinoma, and non-Hodgkin’s lymphoma. Moreover, HCL was diagnosed as a second primary cancer in 97 patients with a previous neoplasm; the relative risk of HCL was 1.62, which was particularly significant after prostate cancer and non-Hodgkin’s lymphoma. Even though information about HCL treatment and overall outcome was not reported, the authors postulated a role for immunosuppression in the pathogenesis of the associated cancers. The same authors compared the prognoses of 718 HCL patients with (119, 16.6%) and without (599, 83.4%) a diagnosis of a second cancer during a median follow-up of 7 years [21]. Among the 234 patients who died, 57 (24.4%) had a diagnosis of a second cancer. Even if their second cancer had a good prognosis per se, the patients with HCL and second cancers had significantly worse OS than the patients with HCL and no other neoplasms, although the cause of death was not reported.

In 2014, a multicenter retrospective French survey investigated 487 patients who were variably treated with purine analogs, splenectomy, IFN, and rituximab; among these patients, a second neoplasm was diagnosed in 48 (10%) patients during a median follow-up of 60 months [17]. In particular, 34 (7%) patients developed a solid neoplasm (prostate, lung, skin, or colon), 10 (2%) patients developed an HM (lymphoproliferative neoplasm, myeloma, myelodysplastic syndrome, chronic myelomonocytic leukemia, or acute myeloid leukemia), and 4 (1%) patients had both a solid neoplasm and an HM. The authors reported an excess of second cancer diagnoses (SIR 1.86, 95% CI 1.34–2.51), especially regarding second HMs (SIR 5.32, 95% CI 2.90–8.92).

A single-center investigation of 88 patients aged less than 40 years reported 11 diagnoses of second neoplasms among 8 (9%) patients after a median time from diagnosis of HCL of 301 months (178–395 months) [18]. The authors reported an excess frequency of second malignancies of 1.60 (95% CI 0.80–2.89), although this difference was not statistically significant.

Another single-center study compared 63 patients aged less than 40 years to 286 older patients mostly treated with purine analogs between June 1983 and December 2013 [12]. The authors reported the diagnosis of a second cancer in 14/63 (22%) younger patients and 56/268 (21%) older patients after a median time from HCL diagnosis of 19 years and 4 years, respectively. Skin cancers and visceral cancers accounted for 33% and 21% of all second neoplasms in the younger group, and for 36% and 39% in the older group, respectively. Two therapy-related myeloid neoplasms (t-MNs) were diagnosed in the younger group, while one t-MN and seven lymphoproliferative disorders were found in the older group.

In our population, second neoplasms were diagnosed after a median of 59.9 months (range: 9.2–169.7 months). No patients developed a second cancer when aged less than 40 years, and only 1/209 aged less than 50 years developed a second cancer. We did not observe an excess of second cancers or an increase in the relative risk for a specific cancer. The SIR was 0.86 (95% CI: 0.54–1.30) for males and 1.13 (95% CI: 0.36–2.73) for females, with no statistically significant differences compared to the general population. The overall mortality was 8.8% (43/489) in the whole non-cancer population and 25% (6/24) among the patients with second cancers: as expected, the median OS was markedly worse among these 24 patients.

Although no t-MNs were diagnosed in our population, different reports of acute and chronic leukemia have been published, including a patient who underwent successful allogeneic stem cell transplantation [20,22,23,24,25,26,27,28,29,30].

The different risk factors for the pathogenesis of HCL-related second cancers might be difficult to distinguish. First, a major level of immunosuppression is reported in the first years following treatment, which is probably caused both by alterations in the immune system linked to HCL itself and by the aftermath of therapy. Second, aging is a risk factor for cancer per se, and this might explain the stable risk of developing second neoplasms during the follow-up period. The combination of aging and intrinsic and acquired alterations of the immune system may result in a marked increase in immunosuppression and favor the development of second neoplasms. Third, patients with HCL have a high rate of cure, and the possibility of a longer follow-up period may increase the prevalence of second cancers when compared with that of other HMs. In more recent years, an increased number of diagnoses of second cancer has also emerged among patients with lymphoproliferative neoplasms due to the improved outcome of these diseases.

Although several types of solid tumors and HMs have been reported, it is possible that HCL is more strongly associated with lymphoproliferative neoplasms, as HCL is also diagnosed as a second cancer after lymphoma. Moreover, t-MNs are not frequently diagnosed after treatment for HCL, probably reflecting a low leukemogenic effect of the therapy used for curing these patients.

The major strength of this study is the single-agent treatment for HCL before developing second cancers. In fact, different anticancer drugs may have different consequences on both the deregulation of the immune system and specific tissue impairment. Although the relatively limited period of observation favored a less variable management of the patients, some diagnoses may have been lost, especially among younger patients. As for most of the analyzed studies, retrospective data collection is the most relevant limitation of this study. Some early-stage cancers may have been missed, and some patients may have been lost during follow-up, irrespective of age. Moreover, the lack of an HCL-specific registry may have reduced the possibility of detecting second neoplasms in patients who moved to another center for their treatment. All of these reasons may have also impacted the variable prevalence of hematologic neoplasms and solid cancers.

## 5. Conclusions

Considering the emerging role of targeted therapies in curing HCL, it would be interesting to evaluate whether the risk of developing a second cancer could be modified with the introduction of disease-specific drugs as a therapeutic approach for HCL. A large, international, prospective registry may be useful to definitively answer the questions surrounding the increased prevalence of second cancers during the follow-up period for HCL and the possible role of different treatments (purine analogs and targeted therapies) in the pathogenesis of second cancers.

## Figures and Tables

**Table 1:** Characteristics of the studied patients.

	Sex	Age at HCL Diagnosis	Response to HCL Therapy	Age at Second Cancer Diagnosis	Time from HCL to Second Cancer Diagnosis (Months)	Type of Second Cancer	Therapy for Second Cancer	Status	Cause of Death	OS from HCL (Months)	OS from Second Cancer (Months)
#1	F	72	CR	76	51.02	Smoldering MM	None	Alive		105.02	54.00
#2	M	49	CR	53	40.52	Dysplastic nevus	Surgery	Alive		60.59	20.07
#3	F	59	PR	65	69.02	Breast carcinoma	Surgery, radiotherapy, hormonal therapy	Dead	Infection during HCL	98.49	29.48
#4	M	34	PR	61	324.33	Villous adenoma colon	Surgery	Alive		409.21	84.89
#5	M	77	CR	82	53.93	DLBCL	Chemotherapy	Dead	Progression of lymphoma	54.66	0.72
#6	M	70	CR	73	33.15	Esophagus carcinoma	Surgery	Alive		38.39	5.25
#7	M	73	HR with no BM evaluation	78	62.85	Basocellular carcinoma	Surgery	Alive		81.18	18.33
#8	M	43	CR	44	13.25	Pleomorphic adenoma	Surgery	Alive		115.84	102.59
#9	M	67	CR	77	127.21	Gastric carcinoma	Surgery	Dead	Cardiovascular event	150.36	23.15
#10	M	67	CR	74	80.26	Basocellular carcinoma	Surgery	Alive		115.48	35.21
#11	M	54	CR	57	26.07	MM IgGk smoldering	None	Alive		68.82	42.75
#12	M	56	CR	71	180.16	Prostate and bladder carcinoma	Surgery	Alive		180.92	0.75
#13	M	57	CR	62	63.57	Prostate carcinoma	Surgery	Alive		173.61	110.03
#14	M	59	PR	66	82.79	Kidney carcinoma	Surgery	Alive		82.79	0.00
#15	M	58	PR	64	66.56	Colon carcinoma	Chemotherapy, surgery	Alive		92.33	25.77
#16	M	73	CR	78	58.98	Prostate carcinoma	Surgery	Alive		176.92	117.93
#17	F	47	CR	56	98.16	Breast carcinoma	Surgery, hormonal therapy	Alive		152.46	54.30
#18	M	70	CR	84	161.02	Colon carcinoma	Surgery	Dead	Surgery complication	161.74	0.72
#19	M	74	CR	79	54.07	Lung carcinoma	Radiotherapy, chemotherapy	Dead	Progression of lung cancer	56.10	2.03
#20	M	75	CR	78	29.90	Colon carcinoma	Surgery	Alive		91.57	61.67
#21	M	74	CR	82	103.61	Mantle cell lymphoma	Chemotherapy	Alive		108.10	4.49
#22	F	54	CR	55	9.87	Basocellular carcinoma	Surgery	Alive		59.44	49.57
#23	M	70	CR	75	54.85	Prostate carcinoma	Surgery	Dead	Natural event	64.36	9.51
#24	M	53	HR with no BM evaluation	56	33.05	Kidney carcinoma	Surgery	Alive		129.41	96.36

Legend: CR, complete response; PR, partial response; MM, multiple myeloma; DLBCL, diffuse large B cell lymphoma.

**Table 2:** Prevalence of second cancers among patients with HCL reported in the literature.

Authors	Calendar Years	Patients (*n*)	Previous Treatment	Second Neoplasms (%)	Relative Risk
Kurzrock 1997 [11]		350	IFN, purine analogs	7.4	n.a.
Cheson 1998 [8]	1992–1993	928	Purine analogs, IFN	5.2	n.a.
Getta 2016 [12]	1983–2013	331	IFN, purine analogs	21	n.a.
Au 1998 [13]	1976–1996	117	Splenectomy, IFN, 2cda	21.3	ORR 2.6
Federico 2002 [14]		1022	None, IFN	5	SIR 1.01
Paltiel 2006 [15]	1991–2001	181	2cda	6.6	SIR 1.3
Hisada 2007 [16]	1973–2002	3104	None, 2cda	11.5	SIR 1.24
Cornet 2014 [17]		487	Purine analogs, splenectomy, IFN, RTX	10	SIR 1.86
Rosenberg 2014 [18]	1986–2012	88	Purine analogs, splenectomy, IFN	10	Excess frequency 1.6
Zheng 2019 [19]	1986–2015	823	n.a.	18.2	ORR 1.65
Paillassa 2020 [20]		279	Purine analogs, splenectomy, IFN, RTX	21	SIR 2.22

Legend: n.a., not applicable; ORR, overall response rate; SIR, standardized incidence ratio.

## Data Availability

Data are contained within the article.
